# Triacylglycerol Crystallinity and Emulsion Colloidal Acid Stability Influence In Vitro Digestion Lipolysis and Bioaccessibility of Long-Chain Omega-3 Fatty Acid-Rich Nanoemulsions

**DOI:** 10.3390/foods14213631

**Published:** 2025-10-24

**Authors:** Jessica D. Ulbikas, Saeed Mirzaee Ghazani, Alejandro G. Marangoni, Amanda J. Wright

**Affiliations:** 1 Department of Human Health Sciences, University of Guelph, 50 Stone Road East, Guelph, ON N1G 2W1, Canada; julbikas@uoguelph.ca; 2 Department of Food Science, University of Guelph, 50 Stone Road East, Guelph, ON N1G 2W1, Canada; smirzaee@uoguelph.ca (S.M.G.); amarango@uoguelph.ca (A.G.M.)

**Keywords:** nanoemulsion, omega-3 fatty acids, docosahexaenoic acid, palm lipids, triacyclglycerols, crystallinity, colloidal acid stability, cooling rate, in vitro digestion, bioaccessibility

## Abstract

This study investigated the relationships between emulsion droplet triacylglycerol (TAG) crystallinity and colloidal acid stability on in vitro digestion microstructure, lipolysis, and docosahexaenoic acid (DHA) bioaccessibility. Oil-in-water (o/w) nanoemulsions (20 wt%) composed of 50/50 DHA-rich algal oil with either palm stearin (PS) or olein (PO), and either acid-stable Tween 80 (2.0 wt%; AS) or acid-unstable soy lecithin (2.2 wt%; AU) were fast or slow cooled to 37 °C after microfluidization. Similar particle size distributions and D_3,2_ (~131–142 nm) and D_4,3_ (~208–239 nm) values were achieved. All emulsions were highly electronegative (~−45–70 mV) and differences (*p* < 0.05) were due to emulsifier type, as expected, and cooling rate. Next, emulsions were subjected to INFOGEST in vitro digestion for analysis of intestinal lipolysis by free fatty acid titration and DHA bioaccessibility. As expected, AU emulsions flocculated, forming larger aggregates during the gastric phase. Slower lipolysis was observed for the AU emulsions (*p* < 0.05), attributed to gastric phase aggregation, and lower 2 h lipolysis was observed for the PS emulsions (~74–77%) based on the presence of crystallinity. DHA bioaccessibility was high (~57–88%), especially for the AS emulsions (*p* < 0.05). Therefore, emulsion colloidal acid stability and TAG physical state significantly impacted emulsion gastric microstructure, digestion, and bioaccessibility.

## 1. Introduction

Omega-3 polyunsaturated fatty acids (n-3 PUFA) are beneficial for optimal human cardiovascular and brain health, in addition to protecting against various biological diseases and conditions, including inflammation, atherosclerosis, hypertriglyceridemia, rheumatoid arthritis, and age-related cognitive decline [1,2,3]. Despite the important health benefits of common long chain n-3 PUFAs, i.e., docosahexaenoic acid (DHA, 22:6, n-3) and eicosapentaenoic acid (EPA, 20:5, n-3), bulk oils containing these fatty acids (FA) tend to have low water solubility and oxidative stability, which impact their shelf life, palatability, and bioaccessibility (i.e., solubilization in the digestate aqueous phase within the gastrointestinal tract and ability to act as a precursor for lipid absorption) [4,5]. For example, lipolytic enzymes may inefficiently adsorb to the surface of emulsified lipid droplets containing EPA and DHA, impacting the ability of these molecules to be hydrolyzed, solubilized in intestinal aqueous phase structures (e.g., mixed bile salt micelles), and ultimately absorbed and distributed throughout the body [5,6]. Thus, strategies to promote their delivery are of interest. Encapsulation in colloidal systems, including nanoemulsions, increases the oil–water interfacial area and can therefore promote lipolysis, bioaccessibility, and subsequent absorption of n-3 PUFA and other lipophilic molecules [7,8,9,10,11]. At the same time, the physical properties of emulsions, including the presence and type of any crystallinity, e.g., solid fat content (SFC), FA chain length, droplet size, and the type of emulsifier present, can influence their interfacial area, microstructure, and gastric structuring within the gastrointestinal tract. These factors can potentially alter lipid digestion, bioaccessibility, and postprandial lipemic response [12,13,14,15,16,17]. Specifically, a better understanding of how TAG crystallinity influences digestion and metabolic response is required [13,16,18,19,20].

Emulsion TAG crystallinity can vary in terms of SFC, polymorphism, and crystal size, shape, and distribution, with differences observed based on composition, cooling rate, shear, and type of emulsifier [15,21,22]. Several studies have explored the influence of emulsion lipid physical state (i.e., solid versus liquid) and SFC on in vitro lipid digestion kinetics, gastric structure, and overall lipid digestibility [9,12,20,23,24,25]. For instance, Thilakarathna et al. observed decreased in vitro lipolysis associated with partial coalescence induced by gastric pH in partially crystalline solid emulsions compared to compositionally equivalent undercooled liquid palm stearin emulsions [16]. Subsequent human studies of these same emulsions highlighted that TAG crystallinity differences were associated with meaningful attenuations in postprandial lipemia [26] and satiety [27]. Golding et al. highlighted the critical role of gastric microstructure on lipid absorption profiles by manipulating the structural stability of emulsions [18]. Also working with algal oil emulsions, Zhou et al. showed that smaller nanoemulsion droplet sizes were associated with increased DHA digestibility and absorption [11]. Lin et al. observed differences in FA bioaccessibility based on in vitro gastric microstructure [14], and Karthik & Anandharamakrishnan also observed higher digestibility for DHA-containing acid-stable versus acid-unstable emulsions [28]. Together, these findings raise the possibility of using emulsion droplet crystallinity, combined with emulsifier type, to tailor digestate microstructure in ways that promote the absorption of biologically important esterified FA, e.g., DHA.

Therefore, this study compared liquid versus partially crystalline emulsion droplets as a strategy to modify emulsion properties and gastrointestinal behaviour. A secondary objective was to explore the potential for cooling rate to influence TAG crystallinity in ways that could influence DHA bioaccessibility. Slower cooling of lipids tends to favour crystal growth, leading to the production of fewer but larger TAG crystals arranged in a more stable lattice form. In contrast, a relatively faster cooling rate tends to favour nucleation, leading to a more rigid, smaller, and less stable TAG crystal network [22,29,30,31]. Cooling rate has not been previously explored as a strategy to alter emulsion crystallinity for lipid delivery. Thus, this research aimed to investigate how different combinations of TAG crystallinity (achieved using liquid versus partially crystalline palm-based lipids and distinct cooling rates) and colloidal acid stability (achieved by different emulsifiers) impact nanoemulsion in vitro digestion behaviour and DHA bioaccessibility. Emulsions with similar baseline droplet size distributions were compared based on being entirely liquid (palm olein (PO)) or partially crystalline (palm stearin (PS)). The hot emulsions were tempered after production using either a slow (SC) or fast (FC) cooling protocol. Based on the emulsifier type, droplets were either acid-unstable (AU), designed to flocculate during the in vitro digestion gastric phase, or were acid-stable (AS) and remained dispersed. Overall, it was postulated that droplet susceptibility to acidic flocculation and partial coalescence in the presence of TAG crystallinity would influence gastric microstructure and thus the interfacial area available for intestinal lipolysis and DHA bioaccessibility. It was specifically hypothesized that the PO–AS emulsions (i.e., PO–AS–SC and PO–AS–FC) would retain a relatively small droplet size during digestion, thus facilitating lipolysis and DHA bioaccessibility.

## 2. Materials and Methods

Materials. Commercially refined PO and PS were purchased from Jewards International (Braintree, MA, USA) and Bunge Oils Inc. (Bradley, IL, USA), respectively, and contained ~45% oleic acid and ~36% palmitic acid, and ~58% palmitic acid and ~27% oleic acid, respectively [21]. DHA-rich algal oil (Life’sDHA^®^) was purchased from DSM (Heerlen, Limburg, The Netherlands), with 359 mg/g as DHA according to the DSM Certificate of Analysis. Tween^®^ 80 (T8) was purchased from Sigma-Aldrich (St. Louis, MO, USA), and liquid soy lecithin (SL) was purchased from NOW Foods (Guelph, ON, Canada). Pepsin from porcine gastric mucosa (P7000), pancreatin from porcine pancreases (8 × USP specifications, including amylase, lipase, protease, ribonuclease, and trypsin; P7545; Sigma-Aldrich, St. Louis, MO, USA), lipase from porcine pancreases (L3126; Sigma-Aldrich, St. Louis, MO, USA), and bile extract (which includes taurine and glycine conjugates of hyodeoxycholic acid and other bile salts; sc-214601; Santa Cruz Biotechnology Inc., Dallas, TX, USA) were used for the in vitro digestion experiments. Hydrochloric acid and sodium hydroxide (1.0 N) (Thermo Fisher Scientific, Waltham, MA, USA) were used along with a pH meter (Accumet Basic AB 15, Thermo Fisher Scientific, Waltham, MA, USA) for pH adjustments during digestion. All other chemicals used were reagent grade. The free fatty acid (FFA) contents of bulk PO (0.25%), PS (0.15%), and algal oil (0.15%), determined by AOCS titration method Ca 5a-40, did not differ (*p* > 0.05).

Emulsion preparation. The eight types of oil-in-water (o/w) emulsions (20 wt% lipid) were prepared with algal oil (10 wt%), either PS or PO (10 wt%), either T8 or SL (2.0 wt% and 2.2 wt%, respectively), and deionized (DI) water. The ingredients were heated for 30 min in a 107 °C oven to erase crystal memory and homogenized with a pre-heated Ultra Turax stand homogenizer (Ika T18 Basic, Willmington, NC, USA) for 1 min at 12,000 rpm. These course emulsions were immediately transferred to a warmed microfluidizer hopper (M110EH, Microfluidics, Westwood, MA, USA), then processed through hot piping with the interaction chamber immersed in an 80 °C water bath for 5 passes at 125 MPa. Samples were collected into glass jars and immediately subjected to one of two cooling protocols. The fast cooling rate involved storage in a 4 °C water bath for 24 h, aiming to induce faster nucleation that would lead to the formation of relatively small TAG crystals. The slow cooling rate involved stepwise cooling in water baths at 60 °C, 40 °C, and 20 °C for 30 min each, then storage in a 4 °C water bath for 24 h. This slow cooling rate aimed to achieve slower nucleation, and thus the presence of relatively few but larger TAG crystals [20,22]. All emulsion analyses were conducted within 7 days of sample preparation, as previous research with these palm lipids indicated that emulsion properties remain unchanged during this timeframe [19,32]. Samples were warmed to 37 °C for 1 h before analyses, and care was taken to limit light and shear exposure during storage and analyses.

In summary, the eight study emulsions, which were 50/50 blends of algal oil with a palm-based lipid, consisted of 4 with PS and 4 with PO (PS or PO). Each blend was stabilized with either an AS or AU emulsifier and cooled after emulsification relatively slowly or quickly (SC or FC).

Particle size distribution (PSD) and zeta potential. Laser diffraction with a Mastersizer 2000S (Malvern Instruments Inc., Southborough, MA, USA) was used to determine PSDs, D_3,2_ values, and D_4,3_ values of each emulsion at baseline. Obscuration values between 12 and 14% and a rotational speed of 1800 rpm were used. Zeta potential, which quantifies droplet electrostatic repulsion, was assessed by a Zetasizer (Zetasizer Nano ZS, Malvern Instruments Inc., Southborough, MA, USA) with sample dilution of 1:250 in DI water. Refractive indices of 1.473 and 1.472 were used for the PO emulsions and PS emulsions, respectively, as determined by a refractometer at 37 °C (Rudolph J357 Automatic Refractometer, Rudolph Research Analytical, Hackettstown, NJ, USA).

Differential scanning calorimetry (DSC) and X-ray diffraction (XRD). The thermal behaviour of all emulsions was investigated with a Mettler-Toledo DSC 1 differential scanning calorimeter (Mettler-Toledo, Columbus, OH, USA) to characterize TAG physical state at baseline. Approximately 20 mg of emulsion was accurately weighed and sealed into hermetic aluminum pans (Mettler-Toledo, Columbus, OH, USA). An empty hermetically sealed aluminum pan was used as the reference. Each sample was equilibrated at 37 °C for 3 min, then heated to 80 °C at a rate of 5 °C/minute to characterize melting behaviour. After holding for 3 min, samples were cooled to 0 °C at 5 °C/minute to characterize recrystallization behaviour. The TA Instrument software (STARe software v1 (2014), Mettler-Toledo, Columbus, OH, USA) was used to characterize the endothermic melting and exothermic recrystallization peaks in terms of the onset and end of transition temperatures. Enthalpy values were also determined. These are directly proportional to the amount of crystalline material present, allowing DSC to be used as a proxy measure of relative TAG crystallinity.

Crystal polymorphism was assessed using a MiniFlex 6G X-ray diffractometer (Rigaku, Tokyo, Japan). Briefly, ~1 mL of sample was placed on a standard sample holder BTS 150 (0.8 mm depth) (Anton Paar, Graz, Styria, Austria) preheated, and held at 37 °C, with scans performed from 15 to 30° at 0.3 °C/minute. The machine was operated at 44 mA and 40 kV with copper-derived X-rays (λ = 1.54 Å) and angle slits of 0.5, 0.5°, and 0.3 mm. Peak positions were calculated using Bragg’s law and SmartLab Studio II (2003) software (Rigaku, Tokyo, Japan). Percent crystallinity of the PS-based emulsions was calculated as the ratio of peak area under the curve values relative to the amorphous background.

Light and confocal laser scanning microscopy (CLSM). Emulsion microstructure was assessed with a light microscope (Olympus, Tokyo, Japan) equipped with a Sony camera (Sony Corporation, Tokyo, Japan). One drop of each emulsion was placed on a glass side and covered with a glass coverslip. Samples were observed under brightfield and polarized conditions at 10×, 20×, 40×, and 100× magnification. Emulsion microstructure was also assessed by CLSM using a Leica TCS SP5 Confocal microscope (Leica Microsystems, Image Acquisition Software AF v2.7, Mannheim, Germany). Nile Red (Sigma-Aldrich, St. Louis, MO, USA) was dissolved in methanol (Sigma-Aldrich, St. Louis, MO, USA) at a 1:1 ratio and added to the samples to achieve a 1:2 ratio of Nile red solution/sample. Samples were gently mixed, incubated at 37 °C for 5 min, placed on cavity well glass microscope slides, and topped with coverslips. Samples were excited at 543 nm with an HeNe laser, and images at 1024 × 1024 pixels were collected using a 63× oil immersion objective lens.

FA composition by gas chromatography (GC). FAs were extracted from the bulk emulsion ingredients (i.e., bulk SL, algal oil, etc.), baseline emulsions, and digestate aqueous phase and methylated to determine FA composition, as previously described [33]. The FA elution peaks were identified by comparing with the retention times of the internal standard C17:0. Data was analyzed using EZChrom Elite version 3.2.1 software (Agilent Technologies, Santa Clara, CA, USA). FA composition was expressed as percent total area count of FA species identified in the GC chromatograms, where FAs greater than 1% were included.

INFOGEST in vitro digestion method. Emulsions were subjected to simulated oral, gastric, and duodenal phases of the INFOGEST (Improving health properties of food by sharing our knowledge on the digestive processes) harmonized static digestion protocol with some minor modifications [34]. NaCl replaced NaHCO_3_ at the same molar ratio to maintain the ionic strength of the fluids and limit pH drift [35]. For the intestinal phase, a pre-determined amount of NaOH (0.25 N) to reach pH 7 was added to the fluids prior to the addition of enzymes. The mixture and a metal stir bar to facilitate mixing were added to a 902 Titrando pH stat (Metrohm, Herisau, Appenzell Ausserrhoden, Switzerland) with a tight-fitting reaction vessel and stir rate of 4 on the Tiamo software (Metrohm, Herisau, Appenzell Ausserrhoden, Switzerland). Pancreatin and lipase solutions (to achieve a trypsin activity of 100 U/mL and a lipase activity of 2000 U/mL in the final mixture, respectively) were added to the reaction vessel at pH 7. Fluids were stirred for a 2 h-long intestinal phase in the pH stat, where the volume of titrant release (0.25 N NaOH) was added against FFA generation and quantified every 15 s. After the intestinal phase, a back titration at pH 9 was performed until 5 min to quantify and correct for FFA that precipitated or solubilized out of solution during intestinal digestion [35,36].

Analyses during in vitro INFOGEST digestion. The volume of titrant release during intestinal digestion was used in the % FFA = 100 × $\frac{V_{NaOH}\times M_{NaOH}\times {MW}_{lipid}}{2\times W_{lipid}}$ equation to determine % FFA release over time for each digestion run, where V_NaOH_ = volume of NaOH required to neutralize FFA release during lipolysis, M_NaOH_ = molarity of NaOH in molar units, MW_lipid_ = molarity of the emulsion lipids, and W_lipid_ = initial weight of emulsion lipid used during in vitro digestion [36]. The volume of titrant (0.25 N NaOH) required during back titration was quantitatively applied to the percent FFA released in the intestinal phase to generate final lipolysis values.

CLSM was also performed on digestate samples to compare emulsion microstructure at key stages during in vitro digestion. These were at the end of the gastric phase of and after the first intestinal pH adjustment.

Lipids solubilized within the aqueous phase of the digestate are considered bioaccessible, thus FA bioaccessibility was determined from the digestate samples. After 2 h in vitro digestion with a back titration, 5 μL/mL (0.5 M) of the lipase inhibitor 4-bromophenylboronic acid (Sigma-Aldrich, St. Louis, MO, USA) dissolved in methanol was added to the digestate. The digestate was then pipetted into tubes and placed in a Ti50 rotor and loaded into the ultracentrifuge (Sorvall WX Ultracentrifuge, Thermo Fisher Scientific, Waltham, MA, USA). Samples were spun at 45,000 rpm and 20 °C for 1 h at 133,718 g force to isolate the aqueous fraction from other digestate components (e.g., precipitates and lipids). The aqueous phase was withdrawn using a fine-tip transfer pipette and stored in a glass borosilicate vial under a nitrogen flush at −20 °C until analysis. FA composition of the aqueous phase samples was determined by GC, as above, and DHA bioaccessibility by weight calculated based on the percent total area count of DHA, weight of lipids added to each in vitro digestion jar, and 2 h lipolysis values.

Data and statistical analyses. Analyses were carried out in triplicate using at least 3 separate emulsion batches, unless otherwise stated. Results are shown as mean ± standard error of the mean (SEM). Analysis of variance (ANOVA) with Tukey’s post hoc testing was performed using GraphPad Prism version 10 for MacOS (GraphPad Software, San Diego, CA, USA) with a significance level of *p* < 0.05. 3-way ANOVA was conducted to examine the effects and interactions of lipids (PS versus PO), emulsifiers (AS versus AU) and cooling rate (SC versus FC). When no effect of cooling rate was observed (*p* > 0.05), data were pooled and 2-way ANOVA conducted. Lastly, a first-order kinetics model (fractional conversional model) [36] was fitted to the in vitro digestion curves to generate the rate constant (k). The model was Y = Y_0_ + (Y_∞_ − Y_0_)(1 − $e^{-Kt}$), where Y = percent FFA release during intestinal lipolysis, Y_0_ = percent lipolysis extent at *t* = 0, *K* = reaction rate constant in min^−1^, and Y_∞_ = asymptomatic value of the curve at *t* = ∞ [37]. In an attempt to improve the curve fitting, a two-phase exponential association model was also fitted to the in vitro digestion curves, generating two rate constants, k_early_ and k_late_, in min^−1^.

## 3. Results and Discussion

### 3.1. Properties of Emulsions at Baseline

#### 3.1.1. Particle Size and Zeta Potential

**Figure 1:**
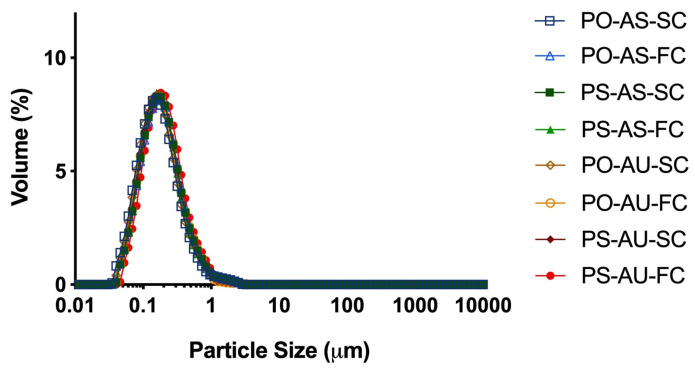
PSD of PO-AS-SC, PO-AS-FC, PS-AS-SC, PS-AS-FC, PO-AU-SC, PO-AU-FC, PS-AU-SC, and PS-AU-FC at baseline. Data reported as mean, *n* = 3.

The PSDs of all emulsions at baseline were similarly monomodal, where all curves overlapped with each other and centered around ~0.13 μm (Figure 1). D_3,2_ and D_4,3_ values ranged between ~131–142 nm and ~208–239 nm, with several small statistical differences observed (*p* < 0.05) (Supplementary Table S1). Since there was no effect of cooling rate (*p* > 0.05) based on 3-way ANOVA, data for the SC and FC emulsions were pooled for a 2-way ANOVA. This showed significant effects of lipid and emulsifier types on D_3,2_ and D_4,3_ values (*p* < 0.05), where generally the AS- and PO-based emulsions had larger particle sizes. Overall, the PSDs of these emulsions were similar to previous work with algal oil SL-stabilized emulsions [32] and smaller than for PS- and PO-based emulsions without algal oil [20]. They were also similar to each other at baseline, which was intended so that differences in interfacial area would not confound the focus on emulsifier type and TAG crystallinity.

All emulsions possessed highly negative zeta potential values (~−43 to −75 mV), where a significant effect of emulsifier were observed (*p* < 0.05), as expected. Differences were also observed based on cooling rate (*p* < 0.05), where the FC emulsions were more electronegatively charged compared to the SC emulsions (i.e., −43.3 ± 0.4 for PO-AS-SC, −49.9 ± 0.3 for PO-AS-FC, −49.5 ± 0.6 for PS-AS-SC, −52.3 ± 0.6 for PS-AS-FC, −71.3 ± 2.2 for PO-AU-SC, −75.2 ± 0.3 for PO-AU-FC, −70.1 ± 0.8 for PS-AU-SC, and −74.3 ± 0.2 for PS-AU-FC). To the best of our knowledge, differences in zeta potential due to cooling rate have not been reported previously. This novel finding warrants further investigation, raising interesting questions about differences in molecular distribution within droplets, including interfacial concentration or expulsion of emulsifying molecules as crystallization proceeds, thereby altering droplet charge. The potential for interfacial differences to influence lipolysis are discussed below.

#### 3.1.2. Emulsion Droplet Crystallinity

**Figure 2:**
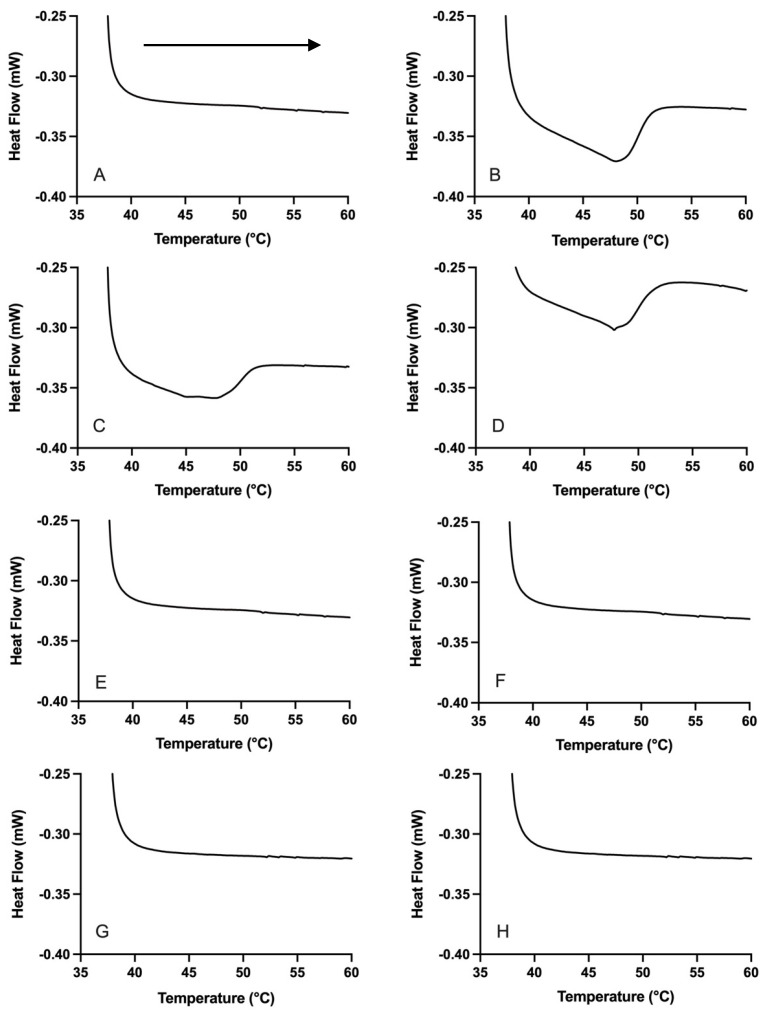
DSC thermograms showing endothermic melting peaks for baseline emulsions PO-AS-SC (**A**), PO-AS-FC (**B**), PS-AS-SC (**C**), PS-AS-FC (**D**), PO-AU-SC (**E**), PO-AU-FC (**F**), PS-AU-SC (**G**), and PS-AU-FC (**H**) during warming in the DSC from 37 °C to 60 °C at 5 °C/min. Arrow indicates direction of temperature (heating). Data reported as mean, *n* = 6.

The DSC melting thermograms for the PO emulsions confirm the absence of crystallinity in these samples (Figure 2) at 37 °C, as intended. Thermal transitions also were not observed in the recrystallization thermograms for these emulsions (Supplementary Figure S1). In contrast, the PS samples possessed crystallinity, evidenced by the presence of endothermic melting peaks (Figure 2). The PS emulsions had similar peak temperatures of recrystallization (*p* > 0.05), but different onset temperatures, end temperatures, and enthalpy values were observed (*p* < 0.05) (Supplementary Table S3). Specifically, the lower onset and end of recrystallization temperatures in the AU emulsions could point to SL delaying nucleation of the emulsified TAG compared to T8. Additionally, the greater diversity of lower-melting FA present in the SL emulsifier compared to the T8 emulsifier could mean different interactions occurred with the PS TAG to alter the recrystallization behaviour. The data in Supplementary Table S3 is evidence of different emulsifier-TAG interactions in the crystalline states of the AS- and AU-PS emulsions.

The absence of XRD peaks in the PO-based emulsions is consistent with the DSC results, confirming the liquid nature of these emulsion droplets (Supplementary Table S4). XRD also highlighted the presence of crystallinity in the PS emulsions, specifically in the form of the β polymorph [38,39]. This is the same as previously reported for similar PS-based emulsions [16,20]. The XRD peak positions for all emulsions were statistically similar (*p* = 0.655). Moreover, there were no statistical differences in terms of the percent crystallinity of samples quantified based on XRD area under the curve values (*p* > 0.05) (Supplementary Table S4). Thus, the XRD data did not support evidence from DSC enthalpy values that a greater amount of crystalline material was present in the SL- (AU) versus T8- (AS) stabilized system following fast cooling (Supplementary Table S4).

#### 3.1.3. Emulsion Droplet Microstructure

**Figure 3:**
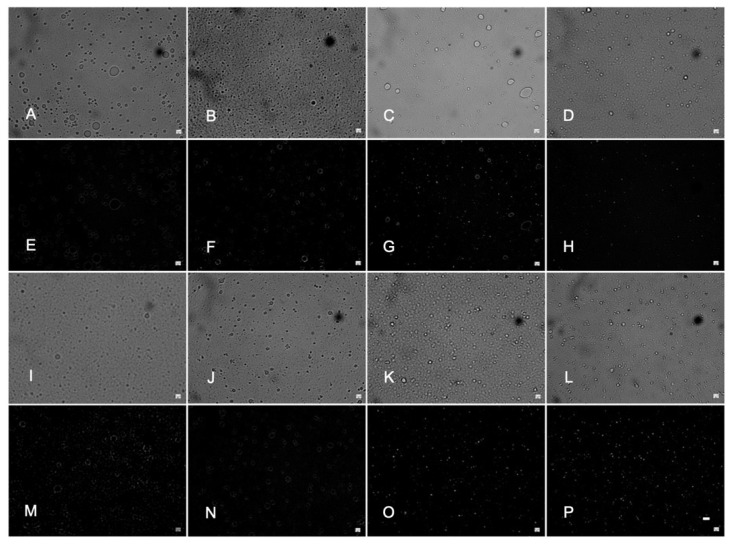
Emulsion microstructure examined under polarized light (**A**–**D**,**I**–**L**) and brightfield (**E**–**H**,**M**–**P**) microscopy: PO-AS-SC (**A**,**E**); PO-AS-FC (**B**,**F**); PS-AS-SC (**C**,**G**); PS-AS-FC (**D**,**H**); PO-AU-SC (**I**,**M**); PO-AU-FC (**J**,**N**); PS-AU-SC (**K**,**O**); and PS-AU-FC (**L**,**P**). Magnification is 100× and the scale bar is 1 μm.

**Figure 4:**
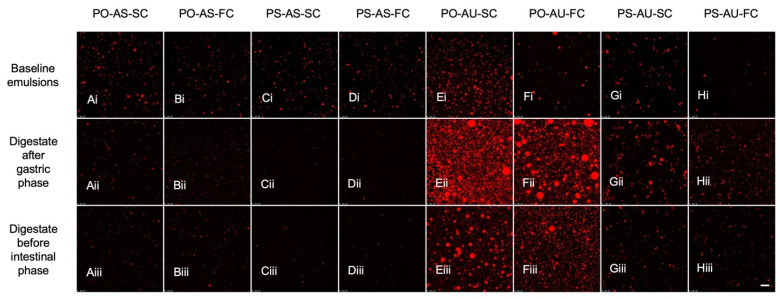
Microstructure of baseline emulsions (i), digestate after the gastric phase (ii), and digestate before the intestinal phase (iii) of in vitro digestion of PO-AS-SC (**A**); PO-AS-FC (**B**); PS-AS-SC (**C**); PS-AS-FC (**D**); PO-AU-SC (**E**); PO-AU-FC (**F**); PS-AU-SC (**G**); and PS-AU-FC (**H**) examined under confocal microscopy. Magnification is 65× and the scale bar is 25 μm.

Emulsion droplet microstructure was assessed using light and CLSM microscopy. According to light microscopy, most visible droplets in the brightfield micrographs were less than 1 μm, although larger structures were occasionally seen, i.e., in PO-AS-SC (Figure 3A) and PS-AS-SC (Figure 3C). Notably, minor aggregation was seen for the AU droplets compared with little to no aggregation in the AS emulsions, although this may have been an artefact of the method. Baseline emulsion microstructure was also analyzed by CLSM (Figure 4). Here, lipid droplets were predominantly spherical and slightly larger for the AU versus AS emulsions. This fits with the SL-stabilized emulsions being more susceptible to flocculation and the light micrograph observations.

CLSM did not suggest differences in emulsion microstructure based on lipid type (PO versus PS). According to PLM, the PO emulsion droplets mostly possessed a white layer around their edges. This phenomenon of organization at the droplet interface was observed by Thilakarathna et al. [16] with Span 60 and liquid canola oil emulsions, although emulsifiers can also interact with TAGs to seed nucleation or even impact polymorphic transitions in some scenarios [40]. In contrast to PO, there was clear evidence that the emulsions based on PS contained crystalline fat in the center of their lipid droplets. Birefringence was observed throughout the core of droplets under polarized conditions (Figure 3G,H,O,P). White dots were more prominent in the SC versus FC droplets (especially PS-AU-SC, Figure 3O), which would be consistent with the slower cooling protocol favouring larger crystal structures. Still, although the SC and FC cooling protocols were intended to manipulate droplet crystallinity, there was minimal to no evidence of this from the baseline emulsion analyses. Lastly, although smaller droplets were at times seen in the brightfield FC emulsion micrographs, this was not consistent with the PSD results where no effect of cooling rate was observed (*p* > 0.05).

### 3.2. Emulsion In Vitro Digestion

#### 3.2.1. Digestate Microstructure

The eight study emulsions were exposed to static in vitro digestion, and CLSM was used to image the microstructure of the digestate after the gastric phase and at the start of the intestinal phase (Figure 4, i.e., after pH adjustment, but before enzyme addition). As expected, flocculation was evident for the AU emulsions, i.e., larger structures were observed during the gastric phase, while little to no droplet aggregation was observed for the T8-based (AS) emulsions. All emulsions were charge-stabilized, although the acidic gastric environment neutralized the SL charges but did not influence T8 ionization, as other studies have reported [41].

Similar amounts of aggregation were seen between the PO and PS emulsions during both the gastric and intestinal stages. This was somewhat unexpected for the intestinal phase, since it was anticipated that the presence of crystallinity in the PS emulsions might lead to partial coalescence in the gastric phase, resulting in structures that would resist redispersal when the pH was increased. While it was hypothesized that cooling rate might have altered the nature of droplet crystallinity and thus partial coalescence during digestion, the FC and SC digestate microstructures visualized by CLSM were similar.

#### 3.2.2. TAG Lipolysis and Relationship to Microstructure

**Figure 5:**
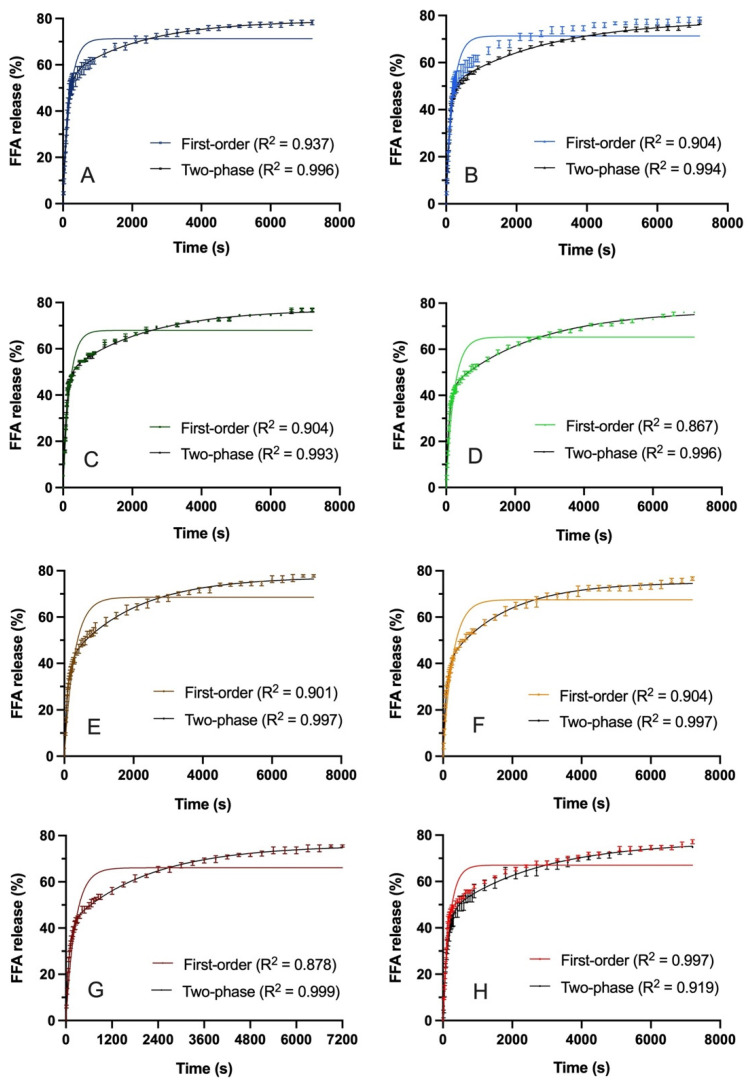
FFA release during in vitro lipolysis in the intestinal phase and first-order kinetics curves for PO-AS-SC (**A**), PO-AS-FC (**B**), PS-AS-SC (**C**), PS-AS-FC (**D**), PO-AU-SC (**E**), PO-AU-FC (**F**), PS-AU-SC (**G**), and PS-AU-FC (**H**). The curves were fitted using the first-order kinetics model and two-phase exponential association, as shown. Data reported as mean ± SEM, *n* = 4.

In vitro intestinal digestions with titration were performed to quantify lipolysis and rate of FFA release for the study emulsions. Data for these experiments are summarized in Figure 5 and Table 1. FFA release was initially rapid and reached near maximum by 2 h for all emulsions. The first-order kinetics model is commonly used to characterize INFOGEST TAG digestion reaction kinetics [42,43]. In this case, R^2^ values in the range of 0.867–0.937 were observed, and the model did not visually fit the data well. Therefore, a two-phase exponential association model was applied and was found to visually fit the data well, with R^2^ values of 0.993–0.999 (Figure 5). For the sake of comparison, the rate constants generated from both models are shown in Table 1, along with the lipolysis values observed at 5 min and 2 h.

Comparing the 5 min lipolysis values shows that early lipolysis was faster for the AS versus AU emulsions (*p* < 0.05). This is consistent with the hypothesis that intestinal phase digestion would be faster when emulsions are not susceptible to gastric flocculation. Effectively, these samples retain a relatively fine droplet microstructure with overall higher interfacial areas for lipase adsorption, i.e., the main parameter determining in vitro lipolysis rate [24]. There were also differences in the first-order rate constants between samples (*p* < 0.05). The primary differences existed between PS-AS-SC and the PO-AU emulsions (0.3289 min^−1^ for PS-AS-SC compared to 0.2125 min^−1^ for PO-AU-FC and 0.2077 min^−1^ for PO-AS-SC). The trend of faster lipolysis for the AS versus AU emulsions mirrors the higher 5 min lipolysis for the AS samples. In contrast, there were no treatment differences observed in k_early_ (or k_late_) from the two-phase exponential model (*p* > 0.05), despite the stronger fits. While the first-order model teased out differences between treatments, the two-phase model did not, even for k_early_, where initial rates of reaction were expected to differ based on the 5 min values. This potentially relates to the high variability in values of k_early_ for replicate digestion runs, given how quickly lipolysis proceeds. In this study, comparing 5 min values was a more robust approach. Others have noted that the first-order model may not be suitable for emulsions with higher lipid content [44,45]. Okuro et al. [45] argued for a more discerning interpretation of in vitro lipolysis and the importance of kinetic modeling that suitably characterizes the reaction kinetics. Although the two-phase model is not typically applied to pH stat lipolysis data, and did not tease out treatment differences in this case, it warrants further consideration given the strong fits and possibility to characterize early versus late-stage kinetics.

Comparing the 2 h lipolysis values based on emulsifier type indicated no significant differences (*p* > 0.05). Thus, colloidal acid stability affected the rate, but not the extent of digestion. Based on lipid physical state (PS versus PO), statistical differences were observed at 2 h (*p* < 0.05). The largest difference was between PO-AS-SC and PS-AU-FC (78.3 ± 1.0% and 74.8 ± 0.4%, respectively) (*p* < 0.05). This trend of facilitated lipolysis for the liquid versus partially crystalline emulsion droplets was hypothesized and is overall consistent with the literature [20,46]. One previous study showed that oleic acid, which is the predominant FA in PO, was digested initially faster and then at a decreased rate compared to stearic acid for which digestion was more gradual [16]. Additionally, higher FA digestibility has been observed with increased FA saturation and shorter chain length, such as between oleic acid, the predominant FA in PO, and palmitic acid, the predominant FA in PS [47].

Aside from their susceptibility to gastric flocculation, the AS and AU emulsions may have differed in other ways, e.g., ease of surfactant displacement and molecular interactions with the emulsified TAG [48]. These differences could influence interfacial interactions during digestion, but also during emulsion production, as suggested by the differences in zeta potential and the DSC recrystallization temperatures discussed above. For example, emulsions stabilized with low molecular weight surfactants, such as T8, have been shown to have limited lipolysis compared to high molecular weight surfactants due to lower stability and strong interfacial displacement of gastric lipase. High molecular weight surfactants, such as SL, have a higher lipolysis extent with higher stability and moderate interfacial gastric lipase displacement [41]. In this study, it is also unknown if T8 and SL formed similar structures at the interface (e.g., monolayers, bilayers) and the degree to which attributes such as interfacial rheological properties may have influenced the lipolysis kinetics [46,47].

According to small deformation experiments with a concentric cylinder geometry (AR2000, TA Instruments, New Castle, DE, USA), the apparent viscosity of the PS-algal oil blend was significantly higher in the presence of an emulsifier (*p* < 0.05), especially T8 (172.5 ± 3.2 mPa·s) versus SL (120.0 ± 2.8 mPa·s) (*p* < 0.05). The apparent viscosity of the PO–algal oil blend did not differ based on the presence or type of emulsifier (29.4 ± 0.3 mPa·s and 28.3 ± 0.4 mPa·s with T8 and SL, respectively; *p* < 0.05). Unfortunately, comparisons of interfacial tension and rheology more specifically were complicated by the solid versus liquid nature of the emulsions. Probing these parameters in future work may help to explain the observed differences. Similarly, the presence of impurities within the emulsifiers or bulk lipids could contribute to different interfacial interactions and properties. PS and PO contained similarly low levels of FFA, suggesting similar contents of partial glycerides within the palm lipids, but other differences were not explored.

#### 3.2.3. DHA Bioaccessibility

**Figure 6:**
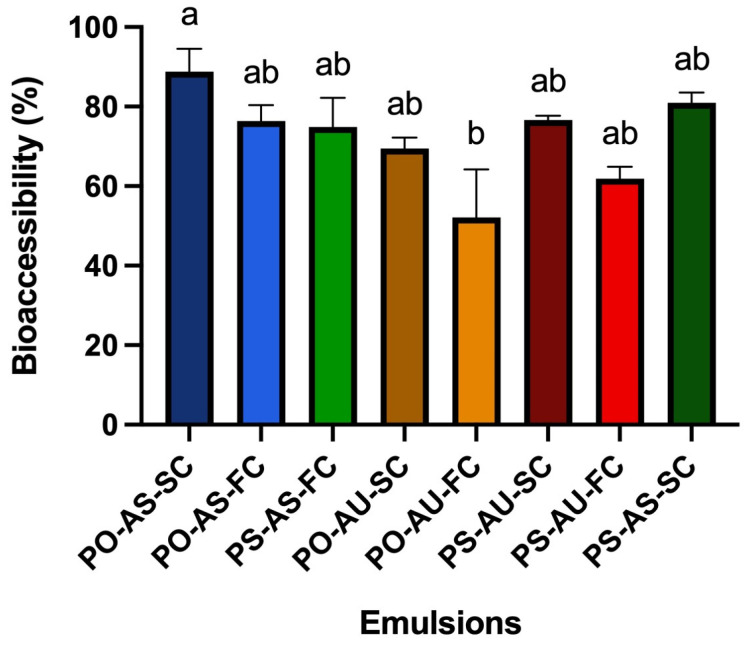
DHA bioaccessibility (i.e., percent of DHA incorporated into the aqueous phase during in vitro digestion) for PO-AS-SC, PO-AS-FC, PS-AS-SC, PS-AS-FC, PO-AU-FC, PO-AU-FC, PS-AU-SC, and PS-AU-FC. Data reported as mean ± SEM; *n* = 6. Different superscript letters “a,b” indicate statistically different values between emulsions; *p* < 0.05.

The percent of DHA incorporated into the aqueous phase during in vitro digestion was compared accounting for the extent of 2 h lipolysis. As seen in Figure 6, DHA bioaccessibility was relatively high for all emulsions, ranging from ~57 to 88%. Not surprisingly, there was no effect of cooling rate (*p* > 0.05). There was a statistically significant difference based on emulsifier type (and a significant lipid–emulsifier interaction; *p* < 0.05), specifically between PO-AS-SC and PS-AU-SC (88.8 ± 5.75% and 57.5 ± 12.55%, respectively), according to the two-way ANOVA (*p* < 0.05). Thus, DHA was more bioaccessible from the AS versus AU emulsions. This aligns with the higher 2 h lipolysis values for the AS emulsions. Since the lipid droplets were smaller prior to intestinal digestion (confirmed by confocal microscopy), there was more interfacial area for lipase absorption and activity in the AS samples. Thus, emulsifier type significantly altered droplet colloidal state and gastric phase structure, which in turn impacted in vitro lipolysis and DHA bioaccessibility.

Notably, DHA bioaccessibility in this work was in the range observed for a previous study of an SL-stabilized emulsion containing the same algal oil, which had higher DHA bioaccessibility (62.49 ± 2.14%) than bulk algal oil and an unhomogenized mixture of algal oil, water, and SL [14]. In another study, DHA bioaccessibility for an oil-in-water emulsion was 71% compared to 58% for bulk algal oil [5], also in the range of the DHA bioaccessibility seen in this work. These trends are expected given what is known about how emulsification enhances lipid digestion and bioaccessibility. In this study, TAG crystallinity did not impact DHA bioaccessibility, but the type of emulsifier did, and this has implications for the design of food-grade emulsions and lipophilic bioactive delivery.

## 4. Conclusions

The effect of TAG physical properties, specifically TAG crystallinity and colloidal acid stability, on altering nanoemulsion microstructure during digestion and supporting n-3 PUFA bioaccessibility was investigated. The physical properties of palm-based emulsions with D_3,2_ ~135 nm were thoroughly compared, where crystallinity was evident and absent for the PS and PO emulsions, respectively. Attempts were made to modify droplet crystallinity with slow versus fast cooling (SC versus FC), but the protocols did not lead to substantial enough differences to affect digestate microstructure, TAG digestion, or DHA bioaccessibility. Microscopy confirmed acidic flocculation of the AU droplets, and this was associated with lower early lipolysis and first-order, but not two-phase, exponential rate constants (*p* < 0.05). Thus, a fine droplet microstructure without susceptibility to acidic flocculation in the stomach facilitated digestion. DHA bioaccessibility was overall high (~57–88%) and higher for the AS emulsions (*p* < 0.05), highlighting the importance of emulsifier type. Future work should explore the effects of more substantial differences in crystallinity as a strategy to tailor gastric microstructure and thus the delivery of biologically important molecules, including DHA and other long chain PUFAs.

## Figures and Tables

**Table 1:** Values of 5 min and 2 h lipolysis and rate constants, k, which characterize the percent FFA release fit to the first-order kinetics model and two-phase exponential association model for PO-AS-SC, PO-AS-FC, PS-AS-SC, PS-AS-FC, PO-AU-SC, PO-AU-FC, PS-AU-SC, and PS-AU-FC emulsions during simulated upper intestinal digestion ^1,2^.

Emulsion	5 min Lipolysis (%)	2 h Lipolysis (%)	First-Order Rate Constant	Two-Phase Exponential Association Rate Constant	
			k (min^−1^)	k_early_ (min^−1^)	k_late_ (min^−1^)
PO-AS-SC	52.8 ± 3.6 ^a^	78.3 ± 1.0 ^a^	0.313 ± 0.045 ^ab^	0.542 ± 0.067 ^a^	0.0278 ± 0.005 ^a^
PO-AS-FC	49.1 ± 0.78 ^ab^	77.2 ± 0.9 ^ab^	0.317 ± 0.023 ^ab^	0.671 ± 0.045 ^a^	0.027 ± 0.002 ^a^
PS-AS-SC	49.2 ± 0.77 ^ab^	76.6 ± 0.7 ^ab^	0.329 ± 0.017 ^a^	0.630 ± 0.040 ^a^	0.031 ± 0.002 ^a^
PS-AS-FC	43.0 ± 1.49 ^b^	76.2 ± 0.3 ^ab^	0.262 ± 0.016 ^ab^	0.717 ± 0.025 ^a^	0.029 ± 0.000 ^a^
PO-AU-SC	42.5 ± 2.09 ^b^	77.8 ± 0.6 ^ab^	0.208 ± 0.018 ^b^	0.596 ± 0.038 ^a^	0.023 ± 0.003 ^a^
PO-AU-FC	42.6 ± 0.69 ^b^	76.6 ± 0.7 ^ab^	0.213 ± 0.004 ^b^	0.709 ± 0.041 ^a^	0.024 ± 0.002 ^a^
PS-AU-SC	43.7 ± 1.42 ^ab^	75.5 ± 0.5 ^ab^	0.248 ± 0.022 ^ab^	0.643 ± 0.071 ^a^	0.033 ± 0.005 ^a^
PS-AU-FC	44.0 ± 3.26 ^ab^	74.8 ± 0.4 ^b^	0.237 ± 0.031 ^ab^	0.504 ± 0.040 ^a^	0.024 ± 0.000 ^a^

^1^ Data reported as mean ± SEM, *n* = 4. ^2^ Within each column, different superscript letters indicate statistically different values, *p* < 0.05.

## Data Availability

The original contributions presented in the study are included in the article/Supplementary Material, further inquiries can be directed to the corresponding author.

## Supplementary Materials

The following supporting information can be downloaded at https://www.mdpi.com/article/10.3390/foods14213631/s1, Figure S1. DSC recrystallization thermograms showing exothermic peaks for the PO-AS-SC (A), PO-AS-FC (B), PS-AS-SC (C), PS-AS-FC (D), PO-AU-SC (E), PO-AU-FC (F), PS-AU-SC (G), and PS-AU-FC (H) emulsions cooled at 5 °C/min from 60 °C to 0 °C (after melting emulsions in the DSC at 5 °C/min from 37 °C to 80 °C and holding for 3 min). Arrow indicates direction of temperature (cooling). Data reported as mean, *n* = 3; Figure S2. DSC melting (A, C, and E, endothermic peaks) and recrystallization (B, D, and F, exothermic peaks) thermograms, respectively, of bulk algal oil (A and B), bulk T8 (C and D), and bulk SL (E and F) during heating and cooling at 5 °C/min. Data reported as mean, *n* = 3. Arrows indicate direction of temperature profile. Inset in E shows a magnification of the melting profile of bulk SL. Table S1. Summary of particle diameter D3,2 (μm) and D4,3 (μm) for PO-AS-SC, PO-AS-FC, PS-AS-SC, PS-AS-FC, PO-AU-SC, PO-AU-FC, PS-AU-SC, and PS-AU-FC at baseline; Table S2. Endothermic peak melting temperatures, including onset and end of melting temperatures, and enthalpy values based on Figure 2 thermograms for the palm stearin-based emulsions, i.e., PS-AS-SC, PS-AS-FC, PS-AU-SC, and PS-AU-FC emulsions at baseline; Table S3. Exothermic peak recrystallization temperatures, including onset and end of recrystallization temperatures, and enthalpy values based on Figure S1 thermograms for the palm stearin-based emulsions PS-AS-SC, PS-AS-FC, PS-AU-SC, and PS-AU-FC at baseline. Table S4. X-ray diffractogram peak positions of the dominant, first weaker and second weaker peaks, as well as percent crystallinity for the palm-stearin based emulsions PS-AS-SC, PS-AS-FC, PS-AU-SC, and PS-AU-FC.
