# Supplementary material for: Triacylglycerol Crystallinity and Emulsion Colloidal Acid Stability Influence In Vitro Digestion Lipolysis and Bioaccessibility of Long-Chain Omega-3 Fatty Acid-Rich Nanoemulsions
Source: Foods. 2025 Oct 24;14(21):3631. doi: 10.3390/foods14213631 (PMC12609572; doi:10.3390/foods14213631)
Supplement: Supplementary file 1 [file foods-14-03631-s001.zip › foods-3885111-supplementary.pdf]

foods

Article

Triacylglycerol crystallinity and emulsion colloidal acid stability influence *in vitro* digestion lipolysis and bioaccessibility of long-chain omega-3 fatty acid-rich nanoemulsions

Supplementary Materials

Table S1. Summary of particle diameter $D_{3,2}$ (μm) and $D_{4,3}$ (μm) for PO-AS-SC, PO-AS-FC, PS-AS-SC, PS-AS-FC, PO-AU-SC, PO-AU-FC, PS-AU-SC, and PS-AU-FC at baseline.[1,2]

Emulsion	$D_{3,2}$ values (μm)	$D_{4.3}$ values (μm)
PO-AS-FC	0.142 ± 0.000^{a}	0.239 ± 0.000^{a}
PO-AS-SC	0.140 ± 0.001^{b}	0.237 ± 0.001^{a}
PS-AS-SC	0.136 ± 0.000^{c}	0.225 ± 0.001^{b}
PS-AS-FC	0.133 ± 0.000^{de}	0.215 ± 0.001^{c}
PO-AU-SC	0.133 ± 0.001^{de}	0.218 ± 0.001^{c}
PO-AU-FC	0.133 ± 0.000^{de}	0.214 ± 0.000^{c}
PS-AU-SC	0.133 ± 0.000^{d}	0.211 ± 0.000^{c}
PS-AU-FC	0.131 ± 0.001^{de}	0.208 ± 0.001^{d}

[1] Data reported as mean ± SEM, n=3.
[2] Within each column, different superscript letters indicate statistically different values, $p<0.05$.

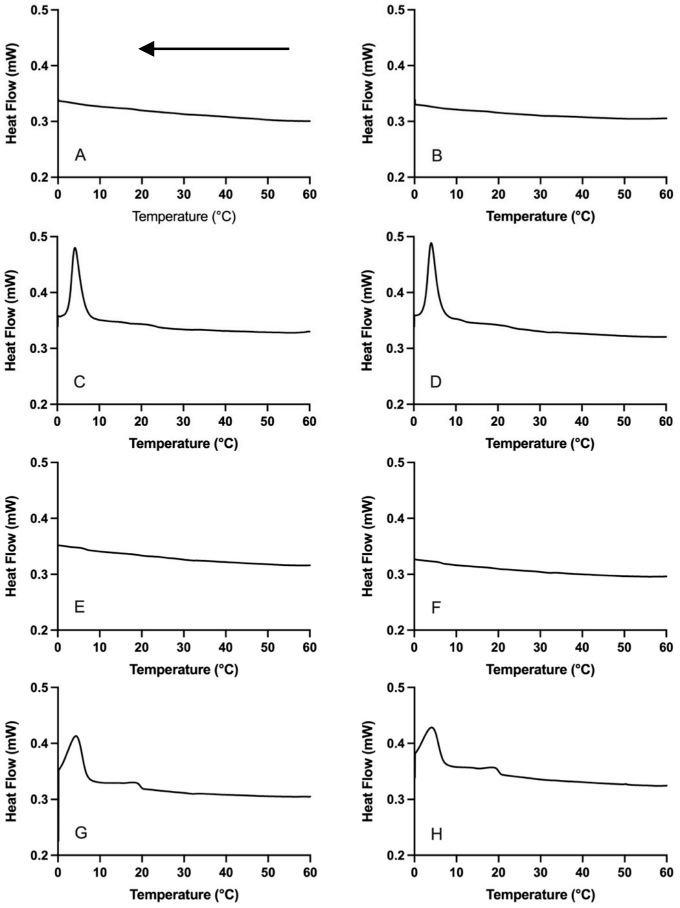

Figure S1. DSC recrystallization thermograms showing exothermic peaks for the PO-AS-SC (A), PO-AS-FC (B), PS-AS-SC (C), PS-AS-FC (D), PO-AU-SC (E), PO-AU-FC (F), PS-AU-SC (G), and PS-AU-FC (H) emulsions cooled at 5°C/minute from 60°C to 0°C (after melting emulsions in the DSC at 5°C/minute from 37°C to 80°C and holding for 3 minutes). Arrow indicates direction of temperature (cooling). Data reported as mean, n=3.

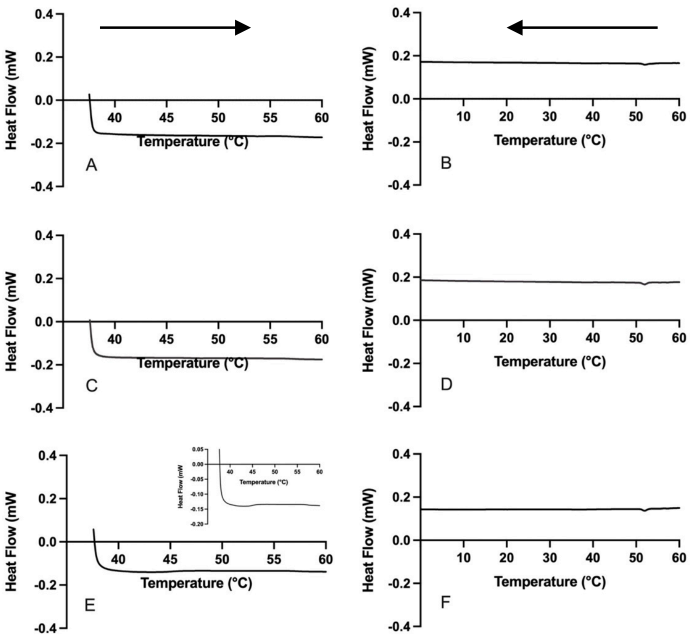

Figure S2. DSC melting (A, C, and E, endothermic peaks) and recrystallization (B, D, and F ,exothermic peaks) thermograms, respectively, of bulk algal oil (A and B), bulk T8 (C and D), and bulk SL (E and F) during heating and cooling at 5°C/minute. Data reported as mean, n=3. Arrows indicate direction of temperature profile. Inset in E shows a magnification of the melting profile of bulk SL.

Table S2. Endothermic peak melting temperatures, including onset and end of melting temperatures, and enthalpy values based on Figure 2 thermograms for the palm stearin-based emulsions, i.e., PS-AS-SC, PS-AS-FC, PS-AU-SC, and PS-AU-FC emulsions at baseline.[1,2,3]

Emulsion	Onset of melting temperature (°C)	Peak of melting temperature (°C)	End of melting temperature (°C)	Enthalpy (J/g)
PS-AS-SC	38.4 ± 0.1^{a}	47.6 ± 0.1^{b}	52.0 ± 0.3^{a}	4.9 ± 0.6^{a}
PS-AS-FC	38.3 ± 0.1^{a}	47.8 ± 0.1^{b}	51.7 ± 0.3^{a}	6.4 ± 2.0^{a}
PS-AU-SC	38.8 ± 0.4^{a}	48.2 ± 0.1^{a}	51.9 ± 0.2^{a}	5.3 ± 0.3^{a}
PS-AU-FC	38.3 ± 0.1^{a}	47.8 ± 0.1^{ab}	52.3 ± 0.3^{a}	5.4 ± 0.3^{a}

[1] Data reported as mean ± SEM, n=6.
[2] Within each column, different superscript letters indicate statistically different values, $p<0.05$.
[3] The emulsions were warmed at 5°C/minute from 37°C to 80°C.

Table S3. Exothermic peak recrystallization temperatures, including onset and end of recrystallization temperatures, and enthalpy values based on Figure S1 thermograms for the palm stearin-based emulsions PS-AS-SC, PS-AS-FC, PS-AU-SC, and PS-AU-FC at baseline.[1,2,3]

Emulsion	Onset of recrystallization temperature (°C)	Peak of recrystallization temperature (°C)	End of recrystallization temperature (°C)	Enthalpy (J/g)
PS-AS-SC	8.5 ± 0.1^{a}	4.3 ± 0.1^{a}	1.7 ± 0.0^{a}	3.8 ± 0.2^{ab}
PS-AS-FC	8.3 ± 0.3^{ab}	4.2 ± 0.1^{a}	1.6 ± 0.1^{a}	4.1 ± 0.1^{a}
PS-AU-SC	7.2 ± 0.4^{b}	4.4 ± 0.1^{a}	0.7 ± 0.1^{b}	3.1 ± 0.2^{bc}
PS-AU-FC	7.9 ± 0.1^{ab}	4.3 ± 0.0^{a}	0.6 ± 0.1^{b}	2.4 ± 0.2^{c}

[1] Data reported as mean ± SEM, n=3.
[2] Within each column, different superscript letters indicate statistically different values, $p<0.05$.
[3] The emulsions were cooled from 80°C to 0°C at 5°C/minute after melting in the DSC at 5°C/minute from 37°C to 80°C.

Table S4. X-ray diffractogram peak positions of the dominant, first weaker, and second weaker peaks, as well as percent crystallinity for the palm-stearin based emulsions PS-AS-SC, PS-AS-FC, PS-AU-SC, and PS-AU-FC.[1,2]

Emulsion	Dominant peak (Å)	First weaker peak (Å)	Second weaker peak (Å)	Percent crystallinity (%)
PS-AS-SC	4.676 ± 0.016[a]	3.955 ± 0.006[a]	3.814 ± 0.005[a]	5.5 ± 0.88[a]
PS-AS-FC	4.654 ± 0.018[a]	3.923 ± 0.023[a]	3.812 ± 0.010[a]	6.1 ± 0.65[a]
PS-AU-SC	4.699 ± 0.014[a]	3.964 ± 0.005[a]	3.823 ± 0.005[a]	6.7 ± 0.78[a]
PS-AU-FC	4.663 ± 0.017[a]	3.949 ± 0.010[a]	3.821 ± 0.005[a]	6.8 ± 0.76[a]

[1] Data reported as mean ± SEM, n=3.

[2] Within each column, different superscript letters indicate statistically different values, $p<0.05$.
